# Supplementary material for: Application of an Ultra-Low-Cost Passive Sampler for Light-Absorbing Carbon in Mongolia
Source: Sensors (Basel). 2023 Nov 5;23(21):8977. doi: 10.3390/s23218977 (PMC10647794; doi:10.3390/s23218977)
Supplement: Supplementary file 1 [file sensors-23-08977-s001.zip › sensors-2607679-supplementary.pdf]

Supplemental Information

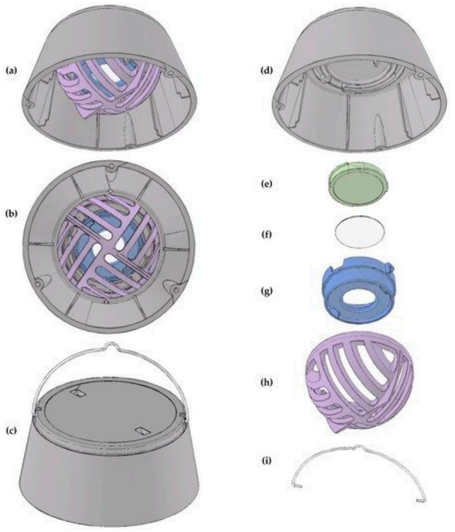

Figure S1. The assembled low-cost passive sampler, shown from (a) side, (b) bottom, and (c) top, and its components, including the (d) cap, (e) base for paper filter exposure surface, (f) paper filter exposure surface, (g) clamp for paper filter exposure surface, (h) protective cage, and (i) wire for hanging sampler. Reproduced from Clark et al. (2020) with permission [16].

Table S1. Deployment and sample details.

Deployment	Dates of deployment	Number of WPS in each household	Number of UPAS in each household	Number of PurpleAir in each household	Number of successful sample (EC-LAC)
1	12/20/2021 - 01/15/2021	2	2	1	2
2	01/15/2021 - 02/10/2021	4	2	1	6
3	02/10/2021 - 03/13/2021	6	2	1	5
4	03/13/2021 - 04/14/2021	8	2	1	8

Table S2. Inter-laboratory comparisons using thermal-optical methods for the determination of EC concentrations.

Study	Number of samples	Number of labs	Protocol	Relative Standard deviation EC (%)	Citation
Birch (1998)	5	5	NIOSH5040	6-26	[37]
Birch (2002)	50	3	NIOSH5040	10	[38]
Schauer et al. (2003)	11	8	ACE-ASIA model	13-21	[39]
Emblico et al. (2012)	14	16	EUSAAR_2, NIOSH	20-25	[40]
Chai et al. (2012)	4	7	NIOSH5040	7-9	[41]
Chiappini et al. (2014)	3	5	NIOSH5040, EUSAAR_2	6.8-19.2	[22]
Panteliadis et al. (2015)	5	17	EUSAAR_2, NIOSH-870	20	[42]
